# Observed Changes of Rain-Season Precipitation in China from 1960 to 2018

**DOI:** 10.3390/ijerph181910031

**Published:** 2021-09-24

**Authors:** Yanyu Zhang, Shuying Zang, Xiangjin Shen, Gaohua Fan

**Affiliations:** 1 Heilongjiang Province Key Laboratory of Geographical Environment Monitoring and Spatial Information Service in Cold Regions, Harbin Normal University, Harbin 150025, China; zyy1827@163.com; 2 Northeast Institute of Geography and Agroecology, Chinese Academy of Sciences, Changchun 130102, China; shenxiangjin@iga.ac.cn; 3 State Key Laboratory of Vegetation and Environmental Change, Institute of Botany, Chinese Academy of Sciences, Beijing 100093, China; fangaohua@ibcas.ac.cn

**Keywords:** precipitation, change, rainy season, precipitation grades, China

## Abstract

Precipitation during the main rain season is important for natural ecosystems and human activities. In this study, according to daily precipitation data from 515 weather stations in China, we analyzed the spatiotemporal variation of rain-season (May–September) precipitation in China from 1960 to 2018. The results showed that rain-season precipitation decreased over China from 1960 to 2018. Rain-season heavy (25 ≤ *p* < 50 mm/day) and very heavy (*p* ≥ 50 mm/day) precipitation showed increasing trends, while rain-season moderate (10 ≤ *p* < 25 mm/day) and light (0.1 ≤ *p* < 10 mm/day) precipitation showed decreasing trends from 1960 to 2018. The temporal changes of precipitation indicated that rain-season light and moderate precipitation displayed downward trends in China from 1980 to 2010 and rain-season heavy and very heavy precipitation showed fluctuant variation from 1960 to 2018. Changes of rain-season precipitation showed clear regional differences. Northwest China and the Tibetan Plateau showed the largest positive trends of precipitation amount and days. In contrast, negative trends were found for almost all precipitation grades in North China Plain, Northeast China, and North Central China. Changes toward drier conditions in these regions probably had a severe impact on agricultural production. In East China, Southeast China and Southwest China, heavy and very heavy precipitation had increased while light and moderate precipitation had decreased. This result implied an increasing risk of flood and mudslides in these regions. The advance in understanding of precipitation change in China will contribute to exactly predict the regional climate change under the background of global climate change.

## 1. Introduction

Precipitation is a main part in the Earth’s hydrological cycle and atmospheric circulation [1,2,3,4]. Changes of precipitation have important impacts on water safety, social economy, ecosystem, and environment [5,6,7,8,9]. Increases in precipitation probably cause more floods, while decreases in precipitation could enhance risks of drought. In recent years, understanding and predicting precipitation change have become an important concern of the government and scientific community in the world [10,11,12]. Precipitation change has obvious regional difference. For example, Bartels et al. [13] showed that precipitation days showed increasing trends in the Midwestern and Northeast United States from 1951 to 2015. Młyński et al. [14] also found increasing trend in the upper vistula basin, Poland. Similarly, there were slightly increasing trends over Slovakia [15]. However, by investigating the changes of precipitation frequent and intensity over 10 hydrological regions in China, Zhang et al. [16] found that the precipitation frequent showed significant downward trend over China from 1956 to 2005. Globally, Westra et al. [17] found that annual maximum daily precipitation showed significant increasing trends for nearly two-thirds of weather stations (5561 observing stations) from 1900 to 2009. Due to the obvious spatial difference of precipitation, understanding the changes of regional precipitation is important for predicting the global climate change.

China has the largest population in the world. The spatiotemporal changes of precipitation over China are very complex due to vast territory [18,19,20,21]. Wu et al. [22] found that precipitation exhibited an increasing trend in the west part of China but decreasing trend in east part of China from 1960 to 2012. Zhang et al. [23] studied the change of precipitation over Yangtze River Basin and indicated that annual mean precipitation days decreased from 1960 to 2005. In addition, in the context of global warming, some studies demonstrated that the extreme precipitation events occurred frequently in China in recent decades [24,25,26,27]. The increasing occurrence of extreme precipitation events is related with the different precipitation grades [28,29]. Many studies suggested that the changes of precipitation with different grades were very complex in different regions of China. For example, Fu et al. [30] found drizzle and small precipitation decreased significantly in South China from 1960 to 2010 due to the increased concentrations of anthropogenic aerosols. In the Yangtze River valley and southeast part of China, Ma et al. [31] demonstrated that light precipitation showed a downward trend while very heavy precipitation showed a upward trend during 1960–2013. In north part of Xinjiang, China, Jiaolan et al. [32] found light, moderate, and heavy precipitation showed significantly increasing trends from 1961 to 2000. Although many studies have investigated the variation of precipitation over China [33,34,35], few studies analyze the changes of precipitation with different grades at both national and regional scales. In the context of global climate change, understanding the changes of precipitation with different grades in different regions of China is important for predicting the regional climate change and guiding the agricultural production in this country.

The precipitation in China mainly occurs from May to September, which significantly influences the agriculture and social economy of this country. On average, precipitation starts over south part of China in May, and moves northward to the north part of China. When the East Asian monsoon ends in August, the rain belt moves back to south part of China in September [36,37,38]. Due to distance to the ocean, rain-season precipitation changes considerably in different regions. In the southeastern part of China, the amount of rain-season total precipitation is the greatest (1000–2000 mm), which is important for growth of rice in these regions. In the northeastern part of China, the precipitation amounts during the rainy season range from 400 to 800. Here, winter wheat, soya beans and corn can grow well in these regions. In west part of China, rain-season total precipitation amounts are the lowest, ranging from 50 to 400 mm. In these regions, the vegetation includes grassland and forest. Understanding the changes of rain-season precipitation in China is important for protecting the ecological environment and guiding agricultural production in China [39,40].

In this study, daily precipitation data from 515 weather stations over China were applied to analyze the spatiotemporal change of rain-season precipitation characteristics (including the number of precipitation days and the precipitation amount) in China from 1960 to 2018. The long-term mean precipitation during the rainy season was first described. Then, the spatiotemporal changes of rain-season precipitation during 1960–2018 were analyzed. Different from previous studies [30,34,41,42,43,44], quality control and homogenization analysis of the daily precipitation data were performed before applying them to study the precipitation change. Furthermore, this study included the longer range of daily precipitation records, which is convenient for comparison of regional results and to better understand the long-term change of precipitation characteristics.

## 2. Data and Methods

### 2.1. Precipitation Data

This study used a data set of daily precipitation, obtained from the China Meteorological Administration (CMA), to analyze the change of rain-season precipitation across China. As the climate data before 1960 contain more gaps, we excluded the data before 1960 and only used the data from 1960 to 2018.

Erroneous outliers may seriously influence the analysis results. Thus, data quality control is very necessary before analyzing the precipitation change. The data quality control was performed by two parts. First, negative daily precipitation values were treated as absent values; second, we also handled daily precipitation values beyond the range of the mean ± 4 times as absent values [45,46].

**Figure 1:**
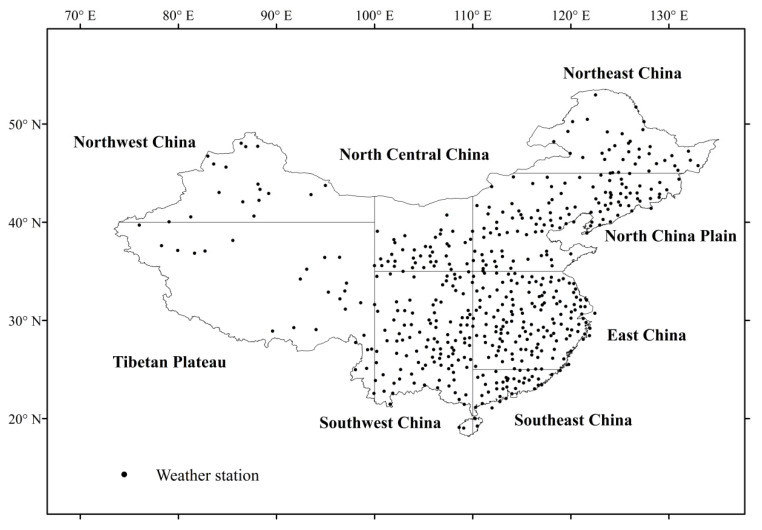
Geographical distribution of the 515 weather stations and eight climatic regions in China.

Data homogeneity analysis is another part that should be considered. When precipitation data are recorded, there are many inevitable changes, such as changes in station location, instrument, observing procedure, and observing environment, etc. These changes may considerably bias the analysis results of precipitation trend. This is why the data homogeneity analysis is essential. After the data quality control, the homogeneity of the precipitation data is checked by the software package RHtests_dlyPrcp (software available at website: http://etccdi.pacificclimate.org/) (accessed on 10 February 2021). According to the penalized maximal *F* test and the penalized maximal *t*, the software package RHtests_dlyPrcp can effectively find out the change points in the time series [47]. Finally, according to the criteria that the missing data is less than 2% of the data series, 515 stations were selected (Figure 1).

### 2.2. Regional Definition and Precipitation Classification

The extent and terrain of China are large and complex, thus the climate over China varies considerably from region to region. Different regions of China may show different climatic features. To better analyze the characteristics of precipitation changes, as defined by the previous studies [48,49], China was divided into eight climatic regions through longitude and latitude in this study (Figure 1). The eight climatic regions can effectively reflect climatic and socioeconomic features over China.

Following Liu et al. [50], a precipitation event during the rainy season is defined as a day with daily precipitation ≥0.1 mm. Based on CMA standards, daily precipitation is classified into four grades: very heavy (*p* ≥ 50 mm), heavy (25 ≤ *p* < 50 mm), moderate (10 ≤ *p* < 25 mm), light (0.1 ≤ *p* < 10 mm) [31,51]. The number of precipitation days and precipitation amount are generally applied to describe the precipitation characteristics in this study. The precipitation amount for each grade is the accumulated precipitation amount within the corresponding interval. The number of precipitation days for each grade is the number of rainy days within the corresponding interval.

### 2.3. Trend Analysis and Statistical Tests

The long-term trends of precipitation characteristics for different grades were evaluated by using the linear regressive method. A linear function was as following

*y* = *at* + *b* (1)

where, *a* is the regression coefficient indicating magnitude of precipitation trend. The rank-based Mann–Kendall was applied to study the statistical significance of the trend, which is generally recommended by the World Meteorological Organization [52]. The rank-based Mann–Kendall is based on the standard normal distribution *Z*

(2) $$Z=\{\begin{array}{c} \frac{S-1}{\sqrt{Var\left(S\right)}}if\;S>0\\ 0\;if\;S=0\\ \frac{S+1}{\sqrt{Var\left(S\right)}}if\;S<0 \end{array}\;$$

in which

(3) $$S=\overset{n-1}{\underset{k=1}{\sum }}\overset{n}{\underset{j=k+1}{\sum }}sgn\left(x_{j}-x_{k}\right)\;$$

(4) $$sgn\left(x_{j}-x_{k}\right)=\{\begin{array}{c} +1\;if\;\left(x_{j}-x_{k}\right)>0\\ 0\;if\;\left(x_{j}-x_{k}\right)=0\\ -1\;if\;\left(x_{j}-x_{k}\right)<0 \end{array}\;$$

(5) $$Var\left(S\right)=\frac{\left[n\left(n-1\right)\left(2n+5\right)-\sum_{i=1}^{m}g_{i}\left(g_{i}-1\right)\left(g_{i}+5\right)\right]}{18}\;$$

where *n* is the number of data points, *m* is the number of tied groups (a tied group is a set of sample data having the same value), and *g_i_* is the number of data points in the *i*th tied group. The advantage of the Mann–Kendall test is that it excludes the assumption of any distribution for the data. In this study, the significant trend was tested at >90% confidence level.

Previous studies found that serial correlation may influent the result of the Mann–Kendall (MK) test by introducing systematic errors [53], thus the influence of series correlation on the Mann–Kendall test should be eliminated before analysis of trend significance. We used the pre-whiten method to remove the serial correlation effects. The lag-1 series correlation coefficient (*r*) was firstly calculated based on Equation (6)

(6) $$r=\frac{\frac{1}{n-1}\sum_{t=1}^{n-1}\left[x_{t}-E\left(x_{t}\right)\right]\left[x_{t+1}-E\left(x_{t}\right)\right]}{\frac{1}{n}\sum_{t=1}^{n}{\left[x_{t}-E\left(x_{t}\right)\right]}^{2}}\;$$

in which

(7) $$E\left(x_{t}\right)=\frac{1}{n}\overset{n}{\underset{t=1}{\sum }}x_{t}\;$$

where *x_t_* is data value at time *t*. If *r* was insignificant at the 95% level (*p* < 0.05), the Mann–Kendall test was applied directly to the original time series of daily precipitation. If *r* was significant at the 95% level (*p* < 0.05), the MK test was used to the “pre-whitened” time series. The “pre-whitened” time series is written as (*x*_2_−*rx*_1_, *x*_3_−*rx*_2_, …, *x*_n_−*rx*_n−1_), where *x*_n_ is the element of the original time series. According to this procedure, the series correlation effect was tested prior to the MK test. The regional or national precipitation index is the average of all stations in the corresponding region. In analyzing temporal change of precipitation characteristics, the nine-point binomial filter was used to smooth out the year-to-year changes of the time series [49].

## 3. Results

### 3.1. Long-Term Mean Precipitation Characteristics during the Rainy Season

**Figure 2:**
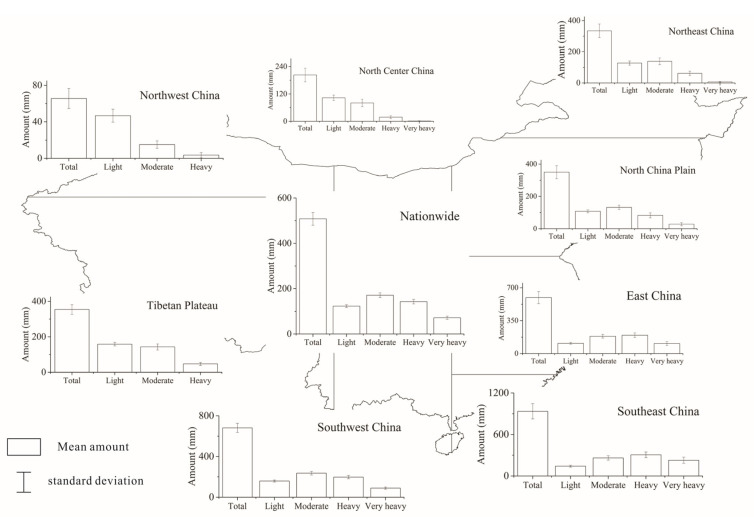
Distribution of long-term (1960–2018) mean amount of rain-season precipitation with different grades.

To better analyze the change of precipitation over China, we analyzed the long-term mean precipitation during the rainy season. The spatial distribution of long-term mean precipitation amount for different precipitation grades during the rainy season is shown in Figure 2. For China as a whole, the long-term (1960–2018) mean amount of precipitation was about 508.07 mm during the rainy season. The distribution of precipitation amount over China showed that the precipitation amount for moderate precipitation was the greatest, accounting for 33.65% of the total precipitation amount during the rainy season. It indicted that moderate precipitation was a main contributor to the total precipitation. The precipitation amounts were quite unevenly distributed across China. During the rainy season of 1960–2018, the total precipitation amount was the largest in Southeast China and decreased westward and northward with the least precipitation amount of 65.59 mm in Northwest China. Similar to the national results, for Northeast China, Southwest China, and North China Plain, moderate precipitation was a main contributor to the total precipitation amount. However, for North Central China, Northwest China, and the Tibetan Plateau, light precipitation had a high contribution to the total precipitation amount. Furthermore, East China and Southeast China had the highest contribution from heavy precipitation.

**Figure 3:**
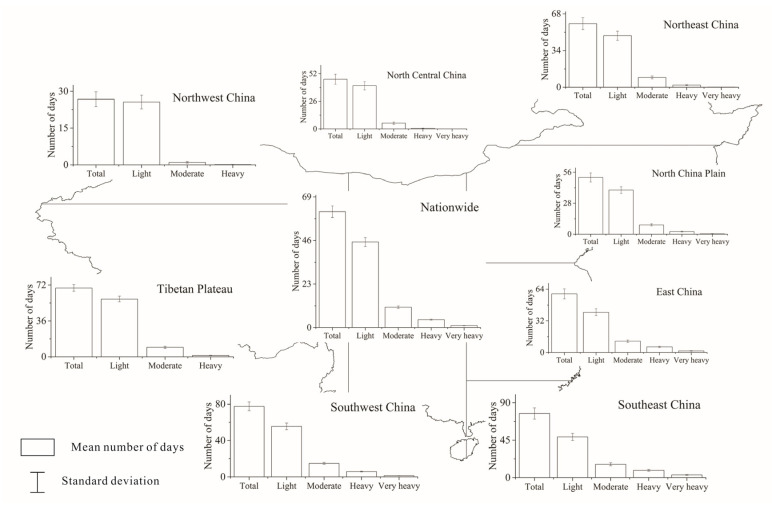
Distribution of long-term (1960–2018) mean number of rain-season precipitation with different grades.

Figure 3 shows the spatial distribution of long-term mean number of precipitation days for different precipitation grades in China and the eight climatic regions during the rainy season of 1960–2018. On average, the number of total precipitation days was about 61 days in China. Different from the distribution of precipitation amount, light precipitation events occurred far more frequently than the other precipitation grades. The light precipitation days accounted for 73.78% of the total precipitation days, which indicated that light precipitation had a high rate of contribution to the total precipitation days. Regionally, the precipitation days were similarly distributed among the eight climatic regions, decreasing exponentially from light precipitation to very heavy precipitation. The region with the greatest precipitation days was located in Southwest China. For Northwest China, the number of heavy precipitation days was the least. Very heavy precipitation is infrequent.

### 3.2. Trends in Precipitation Characteristics during the Rainy Season

**Figure 4:**
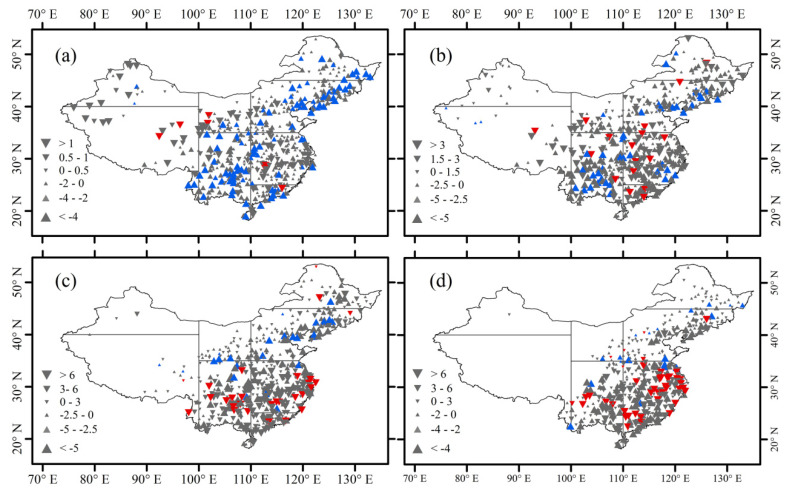
Changes of precipitation amount (mm/decade) ((**a**) light precipitation, (**b**) moderate precipitation, (**c**) heavy precipitation, and (**d**) very heavy precipitation) during the rainy season over China from 1960 to 2018. Upward and downward triangles represent positive and negative trends, respectively. Blue and red triangles denote significant trend at the 90% confidence level.

The trends of rain-season precipitation amount for different precipitation grades in China and the eight climatic regions are shown in Figure 4 and Table 1. At the national scale, the amount of light precipitation decreased significantly at a rate of −1.61 mm/decade from 1960 to 2018 (Table 1). For light precipitation amount, 76.89% of the stations showed negative trends, and 18.64% of the stations showed significant negative trends. At the regional scale, the trends of light precipitation amount were unevenly distributed across China (Figure 4a). The light precipitation amounts mainly showed increasing trends in the west of about 105° E but decreasing trends in the east of about 105° E. As Table 1 shows, we found that the decreasing trend of the light precipitation amount was the greatest in Southwest China. The moderate precipitation amount decreased slightly at a rate of −1.32 mm/decade across China from 1960 to 2018. For moderate precipitation amount, 59.22% of the stations had negative trends, with significant trends for 44 stations (Figure 4b). Regionally, similar to the light precipitation amount, the moderate precipitation amount decreased in most parts of the climatic regions, with significant trends in North China Plain and Southwest China (Table 1). The trends of heavy precipitation amount are shown in Figure 4c. Different from the light and moderate precipitation amount, the heavy precipitation amount showed a slight increase of 0.83 mm/decade during the study period (Table 1). Most stations (49.71%) displayed positive trends, with 28 stations having significant positive trends. Regionally, heavy precipitation showed significant positive trends in the south of about 35° N, and significant negative tendencies in the north of about 35° N. The trends of very heavy precipitation amount are shown in Figure 4d. From 1960 to 2018, the trend of very heavy precipitation amount was 1.23 mm/decade across China. In terms of very heavy precipitation amount, 47.96% of stations displayed positive trends, with significant tendencies at 42 stations (8.16%).

**Figure 5:**
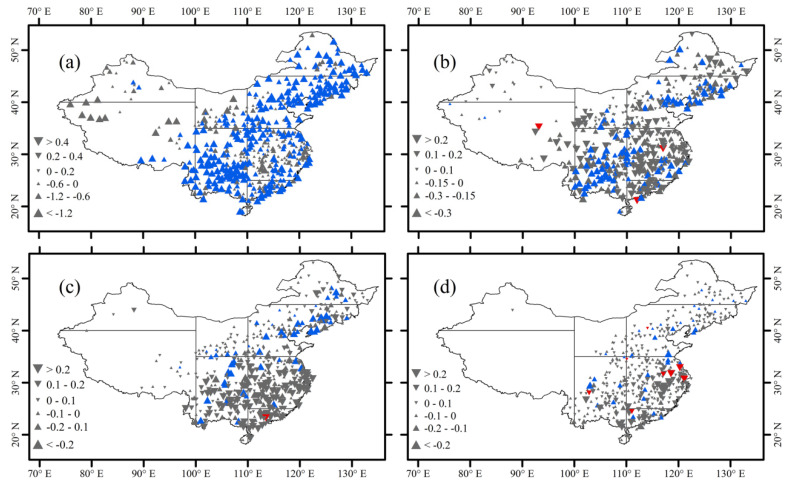
Changes of precipitation days (days/decade) ((**a**) light precipitation, (**b**) moderate precipitation, (**c**) heavy precipitation, and (**d**) very heavy precipitation) during the rainy season over China from 1960 to 2018. Upward and downward triangles represent positive and negative trends, respectively. Blue and red triangles denote significant trend at the 90% confidence level.

Figure 5 and Table 2 show the trends of rain-season precipitation days for different grades in China and the eight climatic regions. The trend of light precipitation was shown in Figure 5a. Nationally, the number of light precipitation days showed a significant decrease of −0.90 days/decade during 1960–2018. The light precipitation days across China decreased at 82.72% of the stations, with the significant decrease at 52.82% of the stations. At the regional scale, most stations showed negative trends. The number of light precipitation days displayed significant trends in the west of about 105° E and insignificant trends in the east of about 105° E. The decreasing trend of light precipitation days was the greatest in Southwest China. Figure 5b shows the trends of moderate precipitation days. For China as a whole, the moderate precipitation days showed a significant decrease of −0.08 days/decade during 1961–2018. The moderate precipitation days decreased at 315 stations (61.17%), and significant trends were found at 89 stations (28.25%). Spatially, similar to the light precipitation amount, the moderate precipitation days decreased in all the climate regions, except in the Tibetan Plateau North Central China, and Northwest China. The trends of heavy precipitation days are shown in Figure 5c. Different from the light and moderate precipitation days, the heavy precipitation days showed an increase of 0.02 days/decade over China from 1960 to 2018. For the heavy precipitation days, 49.51% of stations showed positive trends, and a significant trend was only found at one station. The heavy precipitation days increased considerably in most climatic regions. During 1960–2018, the very heavy precipitation days increased at a rate of 0.02 days/decade over China. For the very heavy precipitation days, 47.96% of stations showed positive trends, and significant trends were found at eight stations (Figure 5d).

### 3.3. Temporal Changes in Precipitation Characteristics during the Rainy Season

**Figure 6:**
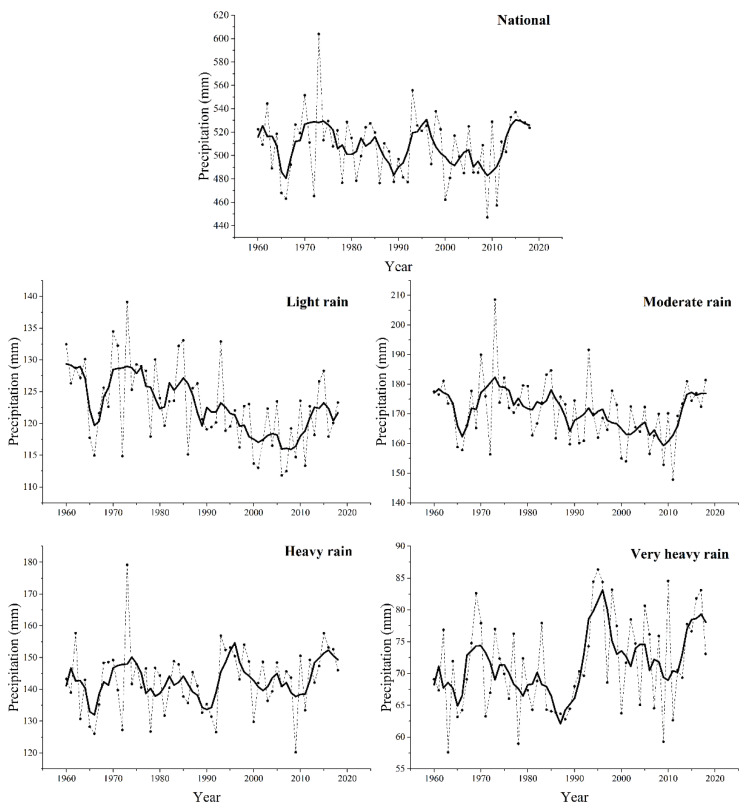
National annual series for precipitation amount during the rainy season in China: total rain-season precipitation, light precipitation, moderate precipitation, heavy precipitation, and very heavy precipitation. The solid line is the result of smoothing with a 9-year binomial filter with reflect ends. The dotted line is national series for precipitation grade.

**Figure 7:**
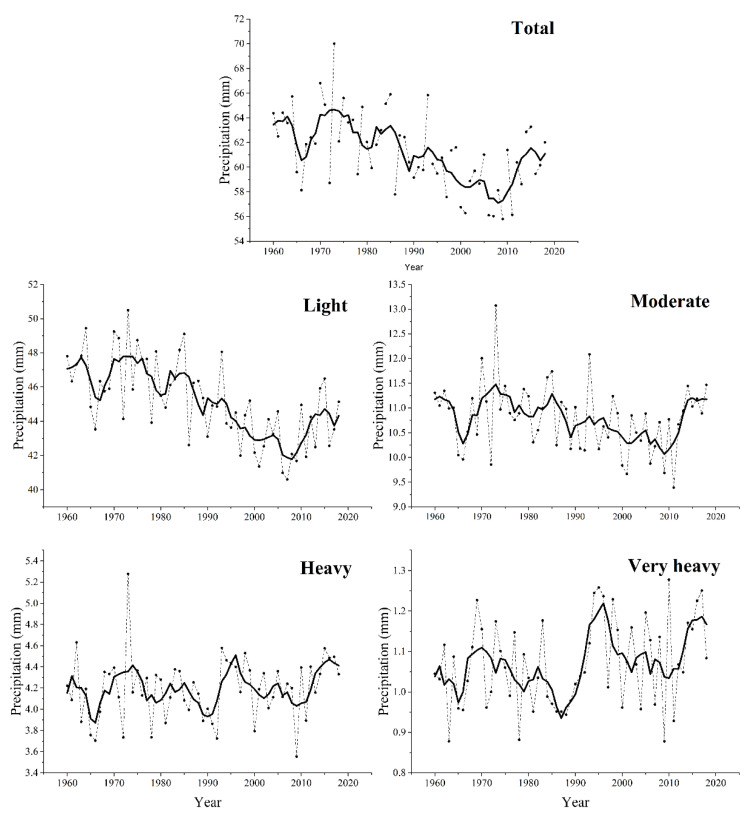
National annual series for number of precipitation days during the rainy season in China: total rain-season precipitation, light precipitation, moderate precipitation, heavy precipitation, and very heavy precipitation. The solid line is the result of smoothing with a 9-year binomial filter with reflect ends. The dotted line is national series for precipitation grade.

In order to further investigate the changes of rain-season precipitation, we analyzed the temporal changes of amounts and days of precipitation with different grades during the rainy season. Figure 6 shows the national annual series of precipitation amount for different precipitation grades during the rainy season of 1960–2018. The temporal change of total precipitation amount showed a large interannual variability during the study period. The light precipitation amount showed no obvious change before the mid-1980s, decreased gradually from the mid-1980s to 2010, and increased after that. There was an obvious decreasing trend of moderate precipitation from 1970 to 2010. Moderate precipitation showed an increasing trend during 2010s. Similar to the total precipitation amount, the heavy precipitation amount had fluctuant variation from 1960 to 2018. For the very heavy precipitation amount, it had fluctuant variation before 1990, and then turned to an increasing trend after 1996 with an obvious peak from 1990 to 2000. Figure 7 shows the national annual series of precipitation days for the different precipitation grades from 1960 to 2018. For the number of total precipitation days, it fluctuated before the mid-1980s, abruptly decreased from 1985 to 2010, and then exhibited an obvious increase after that. Similar to the temporal patterns of precipitation amount, during 1960–1980, the number of precipitation days for all the precipitation grades was the lowest in 1965. From 1980 to 2010, light and moderate precipitation days showed increasing trends. After 2010, all the precipitation grades increased abruptly. Regionally, light precipitation decreased gradually from 1980 to 2010 except in Northwest and the Tibetan Plateau. After 2010, light precipitation increased in all the climatic regions. Very heavy precipitation showed an obvious increasing trend in East China, Southeast China and Southwest China after the mid-1990s (data not shown).

## 4. Discussion

Variability of precipitation during the rainy season directly influences the hydrological cycle and human society. Many precipitation indices were applied to discuss the change of precipitation at the regional and global scales [10,16,31]. In this study, according to the analysis results of number of precipitation days and precipitation amount, we found that precipitation during the rainy season decreased over China from 1960 to 2018. The main reason for this change is that the increase of very heavy precipitation events was much smaller than the decrease of light precipitation events in China during the rainy season. Although very heavy precipitation showed an increasing trend, decreasing light precipitation offset the contribution of increasing very heavy precipitation to the total precipitation during the rainy season. These results were in agreement with the previous finding about an decrease in annual precipitation over China [31]. In other regions of the world, similar results were also found in the Mediterranean, India, Turkey, and East Africa [54,55,56,57]. However, Song, Achberger and Linderholm [41] found that the total amount of rain-season precipitation showed an increasing trend in China from 1961 to 2008. The difference may be attributed to data quality used in this study. Without the quality control and homogeneity analysis, we also found that the total amount of rain-season precipitation showed an increasing trend.

Precipitation during the rainy season showed different trends in different climatic regions. In North China Plain, North Central China, and Northeast China, rain-season precipitation showed decreasing trends. This may be related to the atmospheric circulation change. Many studies showed that the East Asian summer monsoon is weakening significantly during the recent decades [58]. In this context, the northward moisture transport is decreased, causing that moisture supply for precipitation decreased in most parts of northern China. Decreasing precipitation during the rainy season will have a considerable influence on the water resources and availability. Liu et al. [59] pointed out an average water-table decrease of 0.425 m/year in North China Plain from 1983 to 1993, which had a great influence on the environment. This was due to the decreased precipitation amount [60]. Zhang et al. [61] found that severe drought hazard occurred frequently in Northeast China, which had a severe impact on growth of corn.

Different from North Central China, North China Plain, and Northeast China, the analysis results showed that rain-season heavy and very heavy precipitation increased in East China, Southeast China, and Southwest China. However, light and moderate precipitation exhibited decreasing trends in these climatic regions. This indicated that the heavy and very heavy precipitation occurred more frequently than light and moderate precipitation during the rainy season. The implication of this change is that the risk for flood and mudslides during the rainy season increased in these climatic regions. Consequently, socio-economic development was strongly affected. For example, Jiang et al. [62] found more precipitation falls in tense events and significant upward trends in the discharge of the Yangtze River in summer. Wanga et al. [63] found that heavy floods had increasingly occurred in Wujiang River and South China since the 1990s. In the Guangdong province, flood-induced economic loss increased before the 1990s and decreased after that due to enhancing human mitigation and precipitation changes [64]. In addition, this change also leads to the increase of extreme precipitation events in East China, Southeast China and Southwest China. Guan et al. [65] found that, based on 143 meteorological station over the Yangtze River Basin, extreme precipitation events showed a significant upward trend during 1960–2012. Huang et al. [66] explored the spatiotemporal variation of extreme precipitation events in Jiangxi province, China, during 1960–2008 based on nine precipitation indices, and found upward trends of both the days and intensity of extreme precipitation events.

In Northwest China and the Tibetan Plateau, the annual precipitation amounts are less than 400 mm. However, rain-season precipitation displayed increasing trends in these regions. Especially in Northwest China, the precipitation amount and days showed increasing trends. This could be related to climate warming. Previous studies suggested that the change of precipitation is caused by the change of atmospheric moisture content [67,68]. In the context of warming temperature, the water-holding capacity of air increases with the increased atmospheric moisture content [69,70]. Consequently, precipitation increased in these regions. Increasing rain-season precipitation will also influence socio-economic development and environment. Different from the south part of China, the climate in Northwest China and the Tibetan Plateau is rather dry. Therefore, a wet trend may be beneficial to the growth of vegetation and crops in these regions.

## 5. Conclusions

According to daily precipitation data at 515 weather stations over China, the spatiotemporal changes of precipitation amount and number of precipitation days for different precipitation grades in China were analyzed from 1960 to 2018. Nationally, the long-term mean amount and days of rain-season precipitation were about 508.07 mm and 61.24 days, respectively, from 1960 to 2018. The long-term mean total precipitation amount was the largest in Southeast China and the lowest in Northwest China. However, the long-term mean number of total precipitation days was the largest in Southwest China. Both the amount and days of precipitation during the rainy season decreased northward and westward. Light precipitation was a main contributor to the total precipitation amount in the Tibetan Plateau, North Central China, and Northwest China. While, heavy precipitation was a main contributor to the total precipitation in East China and Southeast China.

For the whole of China, the total precipitation amount during the rainy season showed an insignificant decreasing trend from 1960 to 2018. The total number of precipitation days during the rainy season exhibited a significant decreasing trend from 1960 to 2018, with a trend of −0.87 mm/decade. From 1985 to 2010, a clearly downward trend was found according to the nine-point binomial filter method. Very heavy precipitation became more frequent, while the light precipitation decreased significantly over China. Light and moderate precipitation also displayed clear downward trends during 1985–2010. Heavy and very heavy precipitation showed clear interannual changes from 1960 to 2018. Large regional differences existed in the precipitation changes. In Northwest China, both the precipitation amount and days of moderate, heavy, and very heavy precipitation showed downward trends from 1960 to 2018. In Northeast China, North China Plain, and North Central China, almost all precipitation grades displayed downward trends during 1960–2018. In East China, Southwest China, and Southeast China, there was a clear shift from light and moderate precipitation to heavy and very heavy precipitation during the study period.

## Figures and Tables

**Table 1:** Linear trends (mm/decade) of rain-season precipitation amount for different grades in China and its eight climatic regions from 1960 to 2018.

	Total	Light	Moderate	Heavy	Very Heavy
Nationwide	−0.87	−1.61 *	−1.32	0.83	1.23
Northeast China	−3.80	−2.38 *	−1.11	−0.08	−0.24
North China Plain	−7.04 *	−2.32 *	−1.96 *	−2.15 *	−0.62
East China	5.12	−0.96 *	−0.57	2.93 *	3.72 *
Southeast China	4.42	−1.76	−0.68	4.39	2.48
North Central China	−0.73	−0.36	0.13	−0.47 *	−0.02
Southwest China	−4.14	−2.74 *	−3.12 *	0.79	0.93
Northwest China	1.39	0.77	0.24	0.35	–
Tibetan Plateau	0.66	0.12	0.07	0.68	–

* Significant at *p* < 0.1.

**Table 2:** Linear trends (days/decade) of rain-season precipitation days for different grades in China and its eight climatic regions from 1960 to 2018.

	Total	Light	Moderate	Heavy	Very Heavy
Nationwide	−0.90 *	−0.85 *	−0.08 *	0.02	0.02 *
Northeast China	−1.26 *	−1.18 *	−0.07	0.00	−0.01
North China Plain	−1.28 *	−1.07 *	−0.13 *	−0.07 *	−0.01
East China	−0.48 *	−0.06 *	−0.03	0.08 *	0.05 *
Southeast China	−0.91 *	−1.01 *	−0.05	0.12	0.03
North Central China	−0.31	−0.31	0.01	−0.01	0.00
Southwest China	−1.42 *	−1.26 *	−0.20 *	0.02	0.01
Northwest China	0.02	−0.01	0.02	0.01	–
Tibetan Plateau	−0.38 *	−0.42 *	0.03	0.01	–

* Significant at *p* < 0.1.

## Data Availability

The data presented in this study are available on request from the first author.
